# Duration of SARS-CoV-2 mRNA vaccine persistence and factors associated with cardiac involvement in recently vaccinated patients

**DOI:** 10.1038/s41541-023-00742-7

**Published:** 2023-09-27

**Authors:** Aram J. Krauson, Faye Victoria C. Casimero, Zakir Siddiquee, James R. Stone

**Affiliations:** 1 Department of Pathology, Massachusetts General Hospital, Boston, MA USA; 2 Department of Pathology, Harvard Medical School, Boston, MA USA

**Keywords:** Medical research, Diseases

## Abstract

At the start of the COVID-19 pandemic, the BNT162b2 (BioNTech-Pfizer) and mRNA-1273 (Moderna) mRNA vaccines were expediently designed and mass produced. Both vaccines produce the full-length SARS-CoV-2 spike protein for gain of immunity and have greatly reduced mortality and morbidity from SARS-CoV-2 infection. The distribution and duration of SARS-CoV-2 mRNA vaccine persistence in human tissues is unclear. Here, we developed specific RT-qPCR-based assays to detect each mRNA vaccine and screened lymph nodes, liver, spleen, and myocardium from recently vaccinated deceased patients. Vaccine was detected in the axillary lymph nodes in the majority of patients dying within 30 days of vaccination, but not in patients dying more than 30 days from vaccination. Vaccine was not detected in the mediastinal lymph nodes, spleen, or liver. Vaccine was detected in the myocardium in a subset of patients vaccinated within 30 days of death. Cardiac ventricles in which vaccine was detected had healing myocardial injury at the time of vaccination and had more myocardial macrophages than the cardiac ventricles in which vaccine was not detected. These results suggest that SARS-CoV-2 mRNA vaccines routinely persist up to 30 days from vaccination and can be detected in the heart.

## Introduction

**Fig. 1:**
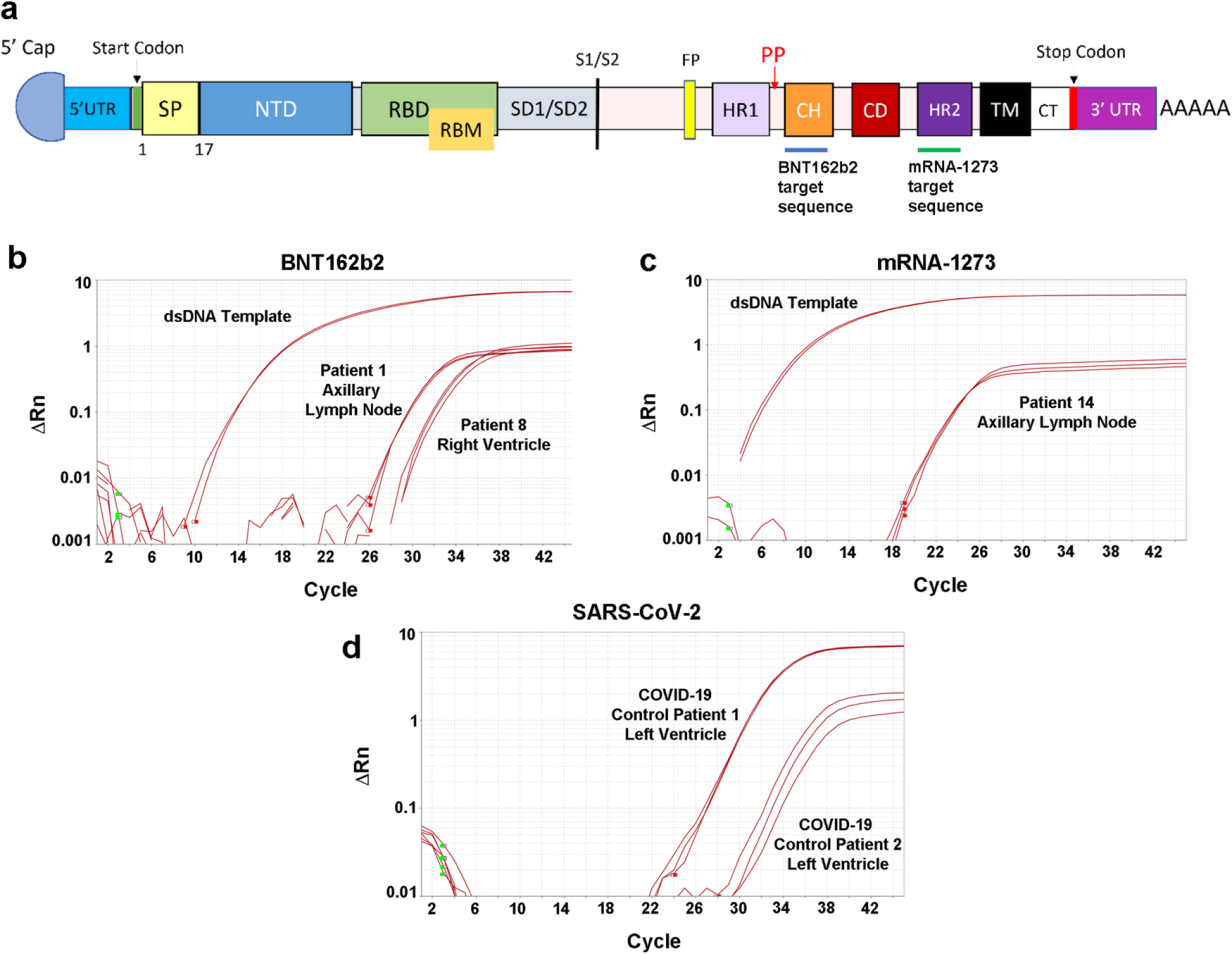
Screening autopsies for mRNA vaccines with designed RT-qPCR assays. Detection of the SARS-CoV-2 mRNA vaccines in autopsies requires devising sequence-specific regions for RT-qPCR assays within the spike gene structure. **a** The functional domains of the mRNA vaccine SARS-CoV-2 spike protein includes the assay targets for the BNT162b2 mRNA vaccine in the central helix (CH) domain (blue line below). For detection of the mRNA-1273 vaccine (green line), the assay detects a sequence within the heptad repeat 2 (HR2) segment (see Table 2). UTR untranslated region, SP signal peptide, NTD N-terminal domain, RBD receptor-binding domain, RBM receptor-binding motif, SD subdomain, FP fusion peptide, HR1 heptad repeat 1, CD connector domain, TM transmembrane, CT cytoplasmic tail. Also marked is the start codon (green line), stop codon (red line), S1/S2 cleavage site (black line), the vaccines’ two Proline substitutions (red P’s), and the polyA tail at the end. Sample amplification plots of RT-qPCR assays to detect **b** BNT162b2 and **c** mRNA-1273 SARS-CoV-2 vaccines in axillary lymph node autopsy samples. Double-stranded DNA templates (dsDNA) were used as positive controls. **d** All vaccine-positive samples were further screened for SARS-CoV-2 E gene. The viral gene was detected only in the two SARS-CoV-2 infected cardiac left ventricle samples (positive controls).

At the start of the COVID-19 pandemic, SARS-CoV-2 vaccines were expediently designed and mass produced, with 12 billion shots administered worldwide by July 2022^1^. In the first year after becoming available, it is estimated that SARS-CoV-2 vaccines prevented 20 million deaths worldwide^2^. Two of the most commonly utilized vaccines are the BNT162b2 (BioNTech-Pfizer) and mRNA-1273 (Moderna) mRNA vaccines, which enable production of full-length SARS-CoV-2 spike protein for gain of immunity (Fig. 1a). Both of these vaccines require a lipid nanoparticle (LNP) for transport. Endosomal-mediated disintegration releases the vaccine mRNA from the LNP for translation by the ribosomes^3^. The two vaccines differ in the composition of the lipids comprising the LNPs^4^. The undesired immunostimulatory response to synthetic mRNA was reduced by substitution of the uridine groups with 1-methyl-3’-pseudouridine^5^. The BNT162b2 and mRNA-1273 mRNA sequences differ from the original SARS-CoV-2 strain with a sequence identity of 72% and 69%, respectively.

The BNT162b2 and mRNA-1273 vaccines have been found to be both relatively safe and effective at preventing severe infection^6,7^. Serious adverse complications due to these vaccines are uncommon and may include anaphylactic reactions, myocarditis, pericarditis, myocardial infarction, cerebral sinus thrombosis, stroke, pulmonary embolism, neuropathies, and autoimmune hepatitis^8–14^. It has been suggested that an understanding of the biodistribution and persistence of the SAR-CoV-2 mRNA vaccines may be critical for gaining insight into some of the rare complications of these vaccines^15^.

The biodistribution and time course of persistence of BNT162b2 and mRNA-1273 in humans are not well understood. In preclinical studies in rats, a radiolabeled LNP-mRNA construct simulating BNT162b2 was detected in most tissues initially, but accumulated primarily at the site of injection and in the liver 48 h after injection^16^. In the same report, using a luciferase-modified LNP-mRNA construct simulating BNT162b2 in mice, protein expression was detected up to 9 days at the injection site, but was not detectable by 48 h in the liver. In similar preclinical studies in rats, a LNP-mRNA construct simulating mRNA-1273, was found to widely distribute to most organs during the first 24 h, but after which primarily localized to the injection site, proximal and distal lymph nodes, and spleen with the half-lives at these sites ranging from 15 to 63 h^17^. The mRNA construct simulating mRNA-1273, was not detected in other tissues beyond 3 days from injection in this animal model.

There have been very few studies assessing the biodistribution and persistence of SARS-CoV-2 mRNA vaccines in humans. Using human axillary lymph node biopsies, spike protein and vaccine mRNA were reported to persist up to 60 days from vaccination with either BNT162b2 or mRNA-1273 as detected by immunohistochemistry and in-situ hybridization^18^. In that study spike protein was also detected in the plasma up to 7 days from vaccination. BNT162b2 mRNA was detected in patients by PCR in circulating leukocytes up to 6 days from vaccination and in the plasma up to 15 days from vaccination^19^. Using highly sensitive single-molecule array assays, spike protein derived from mRNA-1273 was detected in the plasma of patients up to 28 days from most recent vaccination^20^. Circulating exosomes containing spike protein derived from BNT162b2 were detected in patients 4 months after vaccination^21^.

To gain a better understanding of the biodistribution and persistence of SARS-CoV-2 mRNA vaccines, human tissues from autopsies from patients dying after vaccination were assessed for the presence of vaccine by RT-qPCR. The tissues analyzed included not only proximal and distal lymph nodes, but also heart, liver, and spleen. Pathologic features associated with vaccine persistence in the heart were identified. An understanding of the stability of these vaccines in human tissues in combination with patient conditions that contribute to vaccine persistence is important not only for ongoing SARS-CoV-2 vaccinations but for future mRNA vaccine development.

## Results

### Patients analyzed

Tissues were obtained at autopsy from 20 post-vaccinated patients and 5 non-vaccinated control patients. The patient characteristics are shown in Supplementary Table 1. There were no significant differences between the vaccinated and non-vaccinated groups. In none of the vaccinated patients was the cause of death linked to the vaccine (Supplementary Table 1). Seven of the vaccinated patients had received mRNA-1273 and 13 had received BNT162b2. Six of the vaccinated patients received only one vaccine injection, 12 patients received two injections, and two patients received three injections. The intervals between vaccine injections and death are shown in Supplementary Table 2.

### Detection of BNT162b2 and mRNA-1273 vaccines in human tissues

**Fig. 2:**
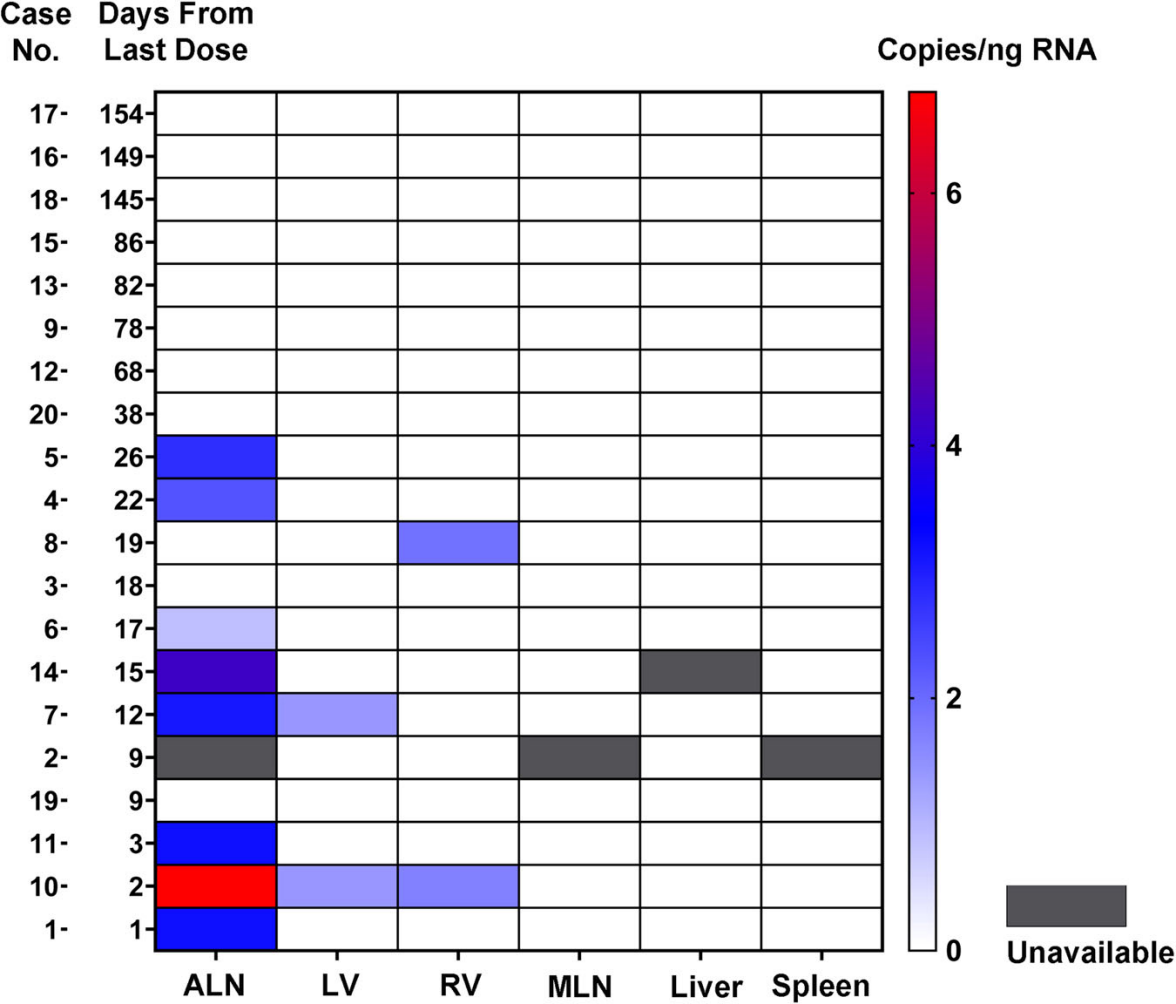
Biodistribution and persistence of SARS-CoV-2 mRNA vaccines. Depicted is a heat map for vaccine detection in the tissues with the patients arranged by the interval in days from last vaccination to death. The scale at the right indicates the copies of vaccine mRNA per ng of RNA. Vaccine mRNA was detected in bilateral axillary lymph nodes (ALN), cardiac left ventricle (LV), and cardiac right ventricle (RV), within 30 days of vaccination in a subset of patients, but not in mediastinal lymph nodes (MLN), liver or spleen.

In the bilateral axillary lymph node samples, vaccine was detected in 2 (33%) of the 6 available samples from patients vaccinated with mRNA-1273 and in 6 (46%) of the 13 patients vaccinated with BNT162b2 (Figs. 1b, c, 2). Overall, for both vaccines, vaccine was detected in 8 (73%) of the 11 available axillary lymph nodes samples from the 12 patients dying within 30 days of vaccination compared with no detection of vaccine in any of the axillary lymph nodes samples from the 8 patients dying after 30 days from vaccination (*P* = 0.003, Fisher exact test). For none of the patients was vaccine detected in the liver, spleen, or mediastinal lymph nodes (each *n* = 19). For the 20 cardiac left ventricle and 20 cardiac right ventricle samples, vaccine was detected in 2 samples of left ventricle and 2 samples of right ventricle from a total of three patients. All three of these patients had been vaccinated with BNT162b2 within 30 days of death. All samples positive for vaccine were validated for sequences outside of the dsDNA control sequence (Supplementary Tables 3–4). Since SARS-CoV-2 can involve tissues throughout the body including the heart in the setting of severe respiratory tract infection^22,23^, as a further control, all tissue samples with detectable vaccine mRNA were screened for the SARS-CoV-2 virus E gene (Fig. 1d) and were found to be negative for the virus. Vaccine was not detected by RT-qPCR in any of the tissues from the 5 non-vaccinated control patients. Immunohistochemistry for the spike protein in the axillary lymph nodes, left ventricle, right ventricle and liver performed on all 20 vaccinated patients showed only non-specific staining (not shown).

### SARS-CoV-2 mRNA vaccine in the heart is associated with myocardial injury

**Fig. 3:**
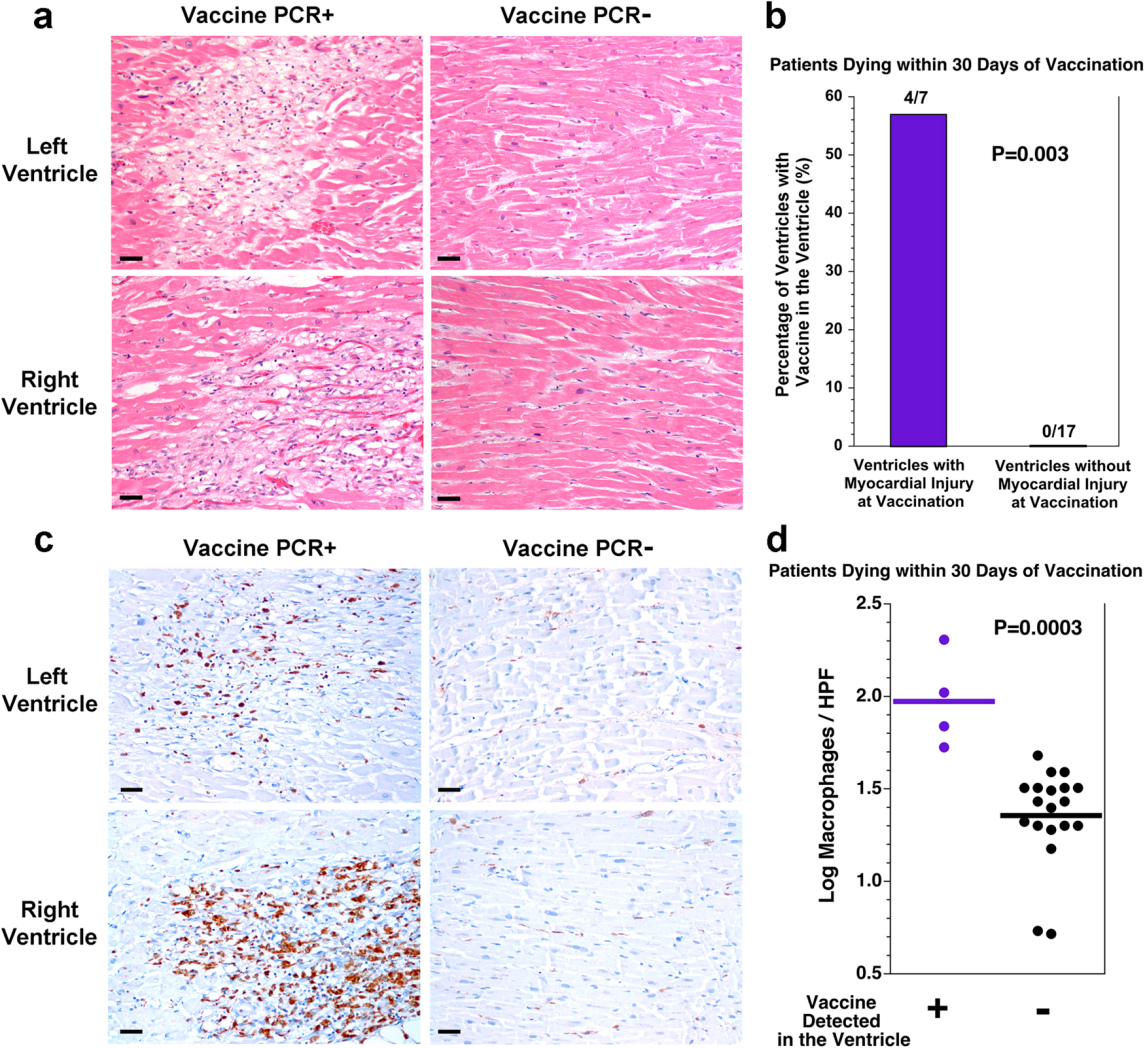
SARS-CoV-2 vaccine in the heart is associated with healing myocardial injury at the time of vaccination and macrophage infiltration in the myocardium. **a** Depicted are histologic images of the myocardium from both the left and right ventricles of the heart with either vaccine detected in the sample or not detected in the sample. For the two samples in which vaccine was detected, there was healing myocardial injury (left), compared with no healing myocardial injury in the two samples in which vaccine was not detected (right). Scale bars indicate 40 microns. **b** For the 12 patients dying within 30 days of vaccination, myocardial injury was present at the time of vaccination in 4 of the 7 ventricular samples with vaccine in the heart but none of the 17 ventricular samples without vaccine in the heart (*P* = 0.003, Fisher exact test). **c** Depicted are immunohistochemical stains for the macrophage marker CD68 on the myocardium from both the left and right ventricles of the heart with either vaccine detected in the sample or not detected in the sample. For the two samples in which vaccine was detected, there are more macrophages (left, brown stain), compared with the two samples in which vaccine was not detected (right). Scale bars indicate 40 microns. **d** Quantitation of the macrophages revealed more macrophages per 400× high power field (HPF) in the ventricular samples in which vaccine was detected than in the ventricular samples in which vaccine was not detected (*P* = 0.0003, *t* test, *n* = 4 vs *n* = 19, *t* = 4.3782, d*f* = 21, difference between means = 0.62, 95% CI: 0.21–0.91). Tissue from one ventricle without vaccine detection and without myocyte injury was not available for CD68 immunohistochemistry.

To gain insight into why some of the patients dying within 30 days of vaccination had detectable vaccine in the heart, the 3 patients with vaccine in the heart were compared with the 9 patients dying within 30 days of vaccination without vaccine in the heart (Table 1). There was no significant difference in age, sex, body mass index (BMI), time since vaccination, or basic cardiac risk factors. While all three of the patients with vaccine in the heart had received BNT162b2, this was not statistically significant. Upon evaluating the histologic slides, none of the patients had myocarditis. However, all three (100%) of the patients with vaccine in the heart had healing myocardial injury which initiated before or at the time of the most recent vaccine injection compared with only 2 (22%) of the 9 patients without vaccine in the heart (Fig. 3a). Considering the specific location of the myocardial injury (left ventricle vs right ventricle) in these patients, vaccine was detected in 4 (57%) of 7 ventricles with myocardial injury at the time of vaccination compared with none (0%) of 17 ventricles without myocardial injury at the time of vaccination (*P* = 0.003, Fisher exact test, Fig. 3b). Healing myocardial injury is associated with macrophage infiltration of the myocardium. Those patients with vaccine in the heart had more macrophages in the myocardium than those patients dying within 30 days of vaccination without vaccine in the heart (Fig. 3c, d, Supplementary Fig. 1, *P* = 0.0003, *t* test).

**Table 1:** Comparison of patients dying within 30 days of vaccination with or without vaccine detected in the heart.

Variable	Vaccine detected in the heart	Vaccine not detected in the heart	*P*
Patients (n)	3	9	
Age (years)^a^	59 ± 1	71 ± 19	0.09^b^
Male sex^c^	2 (67)	4 (44)	1.00^d^
BMI (kg/m^2^)^a^	29 ± 19	24 ± 4	0.69^b^
BNT162b2^c^	3 (100)	5 (56)	0.49^d^
mRNA-1273^c^	0 (0)	4 (44)	
Axillary lymph nodes, vaccine positive^e^	2/3 (67)	6/8 (75)	1.00^d^
Interval from last vaccination to death (days)^a^	11 ± 9	13 ± 8	0.69^b^
History of CAD^e^	0/3 (0)	4/8 (50)	0.24^d^
History of hypertension^e^	1/3 (33)	7/8 (88)	0.15^d^
History of hyperlipidemia^e^	1/3 (33)	6/8 (75)	0.49^d^
History of diabetes mellitus^e^	0/3 (0)	3/8 (38)	0.49^d^
History of autoimmune disease^e^	1/3 (33)	1/8 (13)	0.49^d^
Current or former smoker^e^	1/3 (33)	4/8 (50)	1.00^d^
Post-mortem interval (h)^a^	22 ± 14	25 ± 12	0.76^b^
Heart weight (g)^a^	476 ± 59	430 ± 122 (*n* = 8)	0.43^b^
Left ventricular wall thickness (cm)^a^	1.3 ± 0.3	1.3 ± 0.2	0.81^b^
Focal pericarditis^c^	0 (0)	3 (33)	0.51^d^
Severe CAD (≥75% stenosis)^e^	1/3 (33)	3/8 (38)	1.00^d^
Any acute or recent myocardial injury^c^	3 (100)	6 (67)	0.51^d^
Myocardial injury present at the time of vaccination^c^	3 (100)	2 (22)	0.046^d^

^a^Mean ± SD.

^b^*t* test.

^c^*n* (%).

^d^Fisher exact test.

^e^n/total available (%).

*BMI* body mass index, *CAD* coronary artery disease.

### Assessment of the myocardial injury in the patients with vaccine in the heart

Vaccine was detected in the heart in three patients all of whom had healing myocardial injury. None of these patients showed features of pulmonary thromboembolism or myocardial microthrombotic disease. In patient 7, vaccine was detected in the left ventricle. In this patient, there was myocardial injury in both the left and right ventricles 3–4 weeks old at the time of autopsy, occurring well before the initial vaccine injection 12 days before death. This patient had only moderate coronary artery atherosclerosis and died from an intracranial bleed in the setting of lung cancer. In this patient, the myocardial injury was most likely due to systemic hypoxemia.

In patient 8, vaccine was detected in the right ventricle. This patient had multiple areas of healing myocardial injury in the right ventricle dating from 3–5 days prior to death to 2–3 weeks prior to death, overlapping the time of the second vaccine injection 19 days prior to death. This patient also had only moderate coronary artery atherosclerosis. At autopsy, the lungs displayed pulmonary edema. This patient had a history of heart failure, and died from non-ischemic cardiomyopathy. The right ventricular myocardial injury in this patient was most likely related to right ventricular strain in the setting of heart failure.

In patient 10, vaccine was detected in both ventricles, and this patient had healing myocardial injury dating 3–4 weeks in age in both ventricles. The myocardial injury occurred well before the second vaccine injection 2 days prior to death. The timing of the first vaccine injection is not known. This patient died from severe coronary artery disease, and the myocardial injury at autopsy was most likely related to the coronary artery disease.

## Discussion

The biodistribution and duration of persistence of SARS-CoV-2 mRNA vaccines may be important for understanding some of the uncommon side-effects of these new agents^15^. In this autopsy study, SARS-CoV-2 mRNA vaccines were routinely detected by RT-qPCR in axillary lymph nodes within 30 days of vaccination, in agreement with a previous study analyzing axillary lymph node biopsies by in-situ hybridization^18^. We did not detect vaccine mRNA in mediastinal lymph nodes, consistent with the previous observation that SARS-CoV-2 mRNA vaccines elicit antigen-specific germinal center B cell responses only in draining lymph nodes^24^. Vaccine was not detected by RT-qPCR in the liver or spleen in any of the patients in this study. The absence of detectable vaccine in the liver or spleen was surprising since the liver and spleen had been implicated as sites of vaccine accumulation in pre-clinical rodent studies employing LNPs simulating BNT162b2 and mRNA-1273 respectively^16,17^. However, these pre-clinical rodent studies employed much higher doses of LNPs than are given to patients, potentially explaining the negative results in this study.

An important finding in this study, was the detection of SARS-CoV-2 vaccine mRNA in human heart tissue. Using an RT-qPCR assay, the BNT162b2 vaccine was detected in two left ventricular samples and two right ventricular samples from a total of three patients. Cardiac ventricles in which vaccine was detected had healing myocardial injury at the time of vaccination and had more myocardial macrophages than the cardiac ventricles in which vaccine was not detected. As outlined in the results section, the myocardial injury in these patients was most likely due to the patients’ underlying diseases and not a result of the vaccine itself. It is not known if phagocytic cells transported vaccine mRNA into sites of healing myocardial injury. However, it has been reported that SARS-CoV-2 mRNA vaccines circulate primarily in the plasma compartment rather than the cellular compartment^19^. Another possibility is that the myocardial injury was associated with microvascular permeability changes that allowed circulating vaccine to more efficiently enter the myocardium. Importantly, the liver samples in these patients did not show evidence of similar healing injury. It is also possible that the vaccine LNP’s entered the myocardium by paracellular transport or transcytosis involving intact endothelium in areas of healthy cardiac tissue.

An important adverse complication of SARS-CoV-2 mRNA vaccines is myocarditis^8,9^. While vaccine antigen expression by cardiac myocytes could potentially be a mechanism for myocarditis, we were unable to localize the vaccine antigen by immunohistochemistry, and only observed non-specific staining in both the vaccinated and non-vaccinated control patients. Also, the patients in this study with vaccine detected in the heart were older patients with significant medical conditions, while vaccine-associated myocarditis tends to involve younger patients. None of the patients in this study showed pathologic features of myocarditis. Thus, the relationship of SARS-CoV-2 vaccine distributing to and persisting in the heart and the development of myocarditis is unclear. However, given that SARS-CoV-2 mRNA vaccine was detected in heart muscle with healing injury and that the effects are unclear at the present time, it may be prudent to consider delaying LNP-based vaccination in patients with recent myocardial infarction.

This study has several limitations. Importantly the number of patients with vaccine detected in the heart was very small, making it not possible to definitively determine the reasons vaccine can be detected in the heart in specific patients. This study does not address the specific mechanisms by which vaccine may enter into different organs from the bloodstream. Also, the samples taken for histology and the samples taken for RT-qPCR were random samples; the areas of myocardial injury were microscopic and not visible grossly. Thus, it is not certain that the samples taken for RT-qPCR in the patients with vaccine detected in the heart also contained the myocardial injury present histologically. Only small portions of each organ were examined by RT-qPCR and anti-spike immunohistochemistry, and it is not certain that the tissues sampled are completely representative of the entire organs in terms of the presence of vaccine. Finally, the possibility that variations in post-mortem mRNA degradation between different organs affected the biodistribution of the vaccine observed here cannot be excluded.

In conclusion, this study provides a map of the biodistribution and persistence of SARS-CoV-2 mRNA vaccines in human tissues. A complete understanding of this biodistribution and time course of persistence will be essential as LNP-based vaccines become more widely used for a multitude of pathogens.

## Methods

### Autopsies

Tissues were collected from autopsies performed at Massachusetts General Hospital between January 2021 and February 2022. The tissues collected were bilateral axillary lymph nodes, mediastinal lymph nodes, liver, spleen, cardiac left ventricle, and cardiac right ventricle. Since the location of the injection site of vaccination was usually not known, the axillary lymph nodes were sampled and analyzed bilaterally in all cases. Inclusion criteria were the ability to collect fresh tissue, a clear history of either vaccination or no vaccination, and a post-mortem interval ≤ 60 h. Patients with a history of SARS-CoV-2 infection were excluded from the vaccine group and the non-vaccinated control group. Cardiac tissue from patients dying from SARS-CoV-2 was used as a positive control for SARS-CoV-2 PCR. The study was approved by the Hospital’s Human Subject’s Institutional Review Board (Mass General Brigham IRB). The requirement for patient consent was waived by the IRB, and patient consent was not obtained.

### RNA extraction

Approximately 2 g of tissue was placed into a 50 mL conical tube containing 10 mL TRIzol reagent (Thermo Fisher Scientific, Waltham, MA) and kept at −80 °C. All samples were thawed at room temperature in TRIzol reagent for 10 min to sterilize any SARS-CoV-2 infected samples. Using a Hitachi HVC20 homogenizer (Kinematica AG, Switzerland), tissue was lysed on ice with 5 s durations at high speed, followed by 10+ s at rest.

RNA was extracted from the TRIzol homogenate according to the manufacturer’s protocol. Briefly, 200 µL chloroform was added per 1 mL TRIzol sample and vortexed at high speed for 15 s, and incubated at room temperature for 3 min. The volume of chloroform was increased 2× for samples with visibly high tissue content. After incubation, samples were centrifuged at 12,000*g* for 15 min at 4 °C. Phase-separated RNA was transferred to a clean 1.5 mL tube to which 500 µL of 2-propanol and 4 µL of glycogen were added and mixed by gentle pipetting, followed by 10 min incubation at room temperature. Samples were centrifuged at 12,000*g* for 10 min at 4 °C. The supernatant was aspirated, and pelleted RNA was washed 1–2 times with 75% (v/v) ethanol and spun down at 7500*g* for 5 min at 4 °C. After final aspiration, RNA pellets were briefly air dried, resuspended with 100–400 µL of endonuclease-free water, and placed in a 55 °C heating block for approximately 1 min.

### RNA quality assessment

The yield for each extracted RNA sample was determined using a Nanodrop ND-2000 spectrophotometer (Thermo Fisher Scientific, Waltham, MA). The 280/260 ratio was an initial indicator of each sample’s purity. An EDTA-free protocol from the Joint Genome Institute (JGI)^25^ was applied for all DNase treatment using RN easy kit (Qiagen, Louisville, KY) and 2U of DNAse I enzyme (New England Biolabs, Ipswich, MA). Two-step RT-qPCR assays of housekeeping genes, either with or without the reverse transcriptase (-RT), were performed to further confirm quality RNA yield. The mix with RT contained 0.5–1 µg of total RNA, Random Primers (Promega), Oligo(dT)15 Primer (Promega, Madison, WI), and Superscript III reverse transcriptase (Invitrogen). In triplicate, sample cDNA of 2 µL (10 ng) was added to a 20 µL volume containing SYBR Green Master Mix (Life Technologies, Carlsbad, CA) and 300 nM of each primer (IDT DNA, Coralville, IA, see Supplementary Table 5). Thermocycling settings were 10 min at 95 °C DNA Polymerase activation, followed by 40 cycles with 20 s at 95 °C, 20 s at 60 °C, and 15 s at 72 °C. Amplification *C*_t_ values and melting curve plots of ACTB liver cDNA and ACTB spleen samples (10 ng) were compared to their -RT samples (also 10 ng) in parallel. All other tissue cDNA was compared with -RT controls for GAPDH gene amplification. Extracted RNA used for vaccine detection RT-qPCR experiments had cDNA with *C*_t_ values 7 cycles or higher than -RT parallel mixes.

### RT-qPCR assay for mRNA vaccine detection

The finalized RT-qPCR assay target for BNT162b2 mRNA sequence aligns in most of the central helix domain (CH), whereas for mRNA-1273 it lies within the heptad-repeat-2 domain (HR2) (Fig. 1a). For vaccine-positive controls, double-stranded DNA (dsDNA) fragments of BNT162b2 and mRNA-1273 vaccine sequences were purchased from Integrated DNA Technologies (Coralville, IA). The gBlock^TM^ sequences were selected to either include or be near both vaccines’ two Proline substitutions designed to help stabilize the spike protein (Supplementary Fig. 2)^26,27^. For BNT162b2 detection, primers were designed using Primer3Plus and tested with SYBR Green assay on dsDNA fragments and non-template control (NTC) sample wells. Melting curve analysis was used to determine the best set of primers. Optimized primer concentrations are listed in Table 2. For mRNA-1273 vaccine detection, three sets of primers were tested using serial dilutions against a localized TaqMan probe and the final set selected for optimal primer efficiency score. The primer efficiency of the final BNT162b2 and mRNA-1273 primer/probes sets were 97.7% and 97.2%, respectively (Supplementary Fig. 3). After correcting from dsDNA material in the calibration curves to ssRNA vaccine (i.e. multiplied by 2), the limit of detection (LOD) for BNT162b2 was 6 ssRNA copies/reaction and the LOD for mRNA-1273 was 11 ssRNA copies/reaction.

**Table 2:** Vaccine detection RT-qPCR primer/probe sets.

Target	Primers and probes	Sequence 5’–3’	Final concentration in RT-qPCR (nM)	Amplicon length (bp)
BNT162b2^a^	Forward	GGTGCAGATCGACAGACTGA	400	189
	Reverse	GGGAAGCTCATCAGGTGGTA	400	
	Probe	TACGTGACCCAGCAGCTGATCAGAGCC	200	
mRNA-1273^b^	Forward	AGCTGGACAGCTTCAAGGAG	400	159
	Reverse	GGCTCTCGTTCAGGTTCTTG	400	
	Probe	ACGCCAGCGTGGTGAACATCC	200	

^a^Probe for BNT162b2 was produced as 56-FAM/TACGTGACC/ZEN/CAGCAGCTGATCAGAGCC/3IABkFQ.

^b^Probe for mRNA-1273 was produced as 56-FAM/ACGCCAGCG/ZEN/TGGTGAACATCC/3IABkFQ.

RNA samples of 2 µL (100 ng total) were added to a final volume of 20 µL reaction mixture containing 5 µL TaqMan Fast Virus 1-step Master Mix (Life Technologies, Carlsbad, CA) in triplicate. Thermocycling was performed at 50 °C for 10 min for reverse transcription, followed by 20 s at 95 °C and then 45 cycles of 95 °C for 5 s and 60 °C for 30 s. All assays included dsDNA vaccine sequence fragments (positive controls) and NTC wells (negative controls). Five unvaccinated autopsies were tested with each vaccine assay as additional controls. Criteria for vaccine detection in the assays required samples to have a Ct value below 35 cycles in all three wells tested. As a further control, a SARS-CoV-2 detection assay for *E* gene was administered^28^. Briefly, the same RNA concentration of 100 ng was combined with TaqMan Fast Virus 1-step Master Mix with E Sarbeco primers and probe (IDT DNA, Coralville, IA, see Supplementary Table 5). Incubation for reverse transcription was done at 50 °C for 10 min, followed by 95 °C for 20 s, 45 cycles of 95 °C for 3 s, and 58 °C for 30 s. All fluorescent-based assays were performed on a QuantStudio 3 (Thermo Fisher Scientific).

### Validation of vaccine-positive samples

To validate either BNT162b2- or mRNA-1273-positive samples from the RT-qPCR assays, their cDNA products were used in 1–3 possible PCR reactions to target areas outside the positive control (dsDNA fragment) sequence. Table 3 lists the primer sets, location in the vaccine sequence, and amplicon length. Briefly, PCR reactions were performed with 25 µL reaction mix consisting of 50–100 ng sample cDNA, 1–1.25 µM each primer, and 12.5 µL GoTaq® Green Master Mix (Promega, Madison, WI) following manufacturer’s instructions. Reactions consisted of 40–43 amplification cycles of 30 s denaturation at 95 °C, 60 s annealing at 60 °C, and elongation time dependent of bp length at 72 °C (30 s per 500 bp). PCR products were resolved on 1.5% agarose gel stained with SYBR™ Safe DNA Gel Stain (Thermo Fisher Scientific). Bands of target bp length were excised and purified with QIAquick ® Gel Extraction Kit (Qiagen, Louisville, KY) and sequenced by Genewiz (Cambridge, MA) and analyzed with BLASTn for sequence alignment with the vaccine sequence^29^.

**Table 3:** Validation primer sets.

Target	Sequence location (NT)^a^	Primers	Sequence 5’–3’	Amplicon length (bp)
BNT162b2	60	Forward	CAGAACACAGCTGCCTCCAG	503
		Reverse	TTCTTGAAGTTGCCCTGCTT	
	416	Forward	CCAACGTGGTCATCAAAGTG	1128
		Reverse	AAGCTCAGCACCACCACTCT	
	2969	Forward^b^	GGTGCAGATCGACAGACTGA	682
		Reverse	CAGATGTACCAGGGCCACTT	
mRNA-1273	160	Forward	CTGTTCCTGCCCTTCTTCAGC	405
		Reverse	GGTTCTTGAAGTTGCCCTGCTTG	

^a^Sequence location is numbered in relation to the start codon of the vaccine’s signal peptide segment.

^b^Forward primer is the same as for the assay. The reverse primer extends 480 NTs past the dsDNA control.

### Assessment of the timing of onset of myocardial injury

The age of healing myocardial injury in the specimens was estimated using standard pathologic features: myocardial necrosis with low amounts of neutrophils (days 1–2), large amounts of neutrophils (days 3–5), macrophage infiltration and myocyte removal (days 4–14), loose granulation tissue (days 14–21), late collagenous granulation tissue (days 21–28), and dense scar formation (more than 28 days)^30,31^.

### Immunohistochemistry

Tissue sections from formalin-fixed paraffin-embedded tissue were mounted on positively charged glass slides. Deparaffinization and rehydration of sections were done by treating slides with Xylene (2 treatments 5 min each) and graded ethanol (100, 90, 70, 50%, and water) solutions, respectively. Following rehydration, antigen retrieval was carried out by the heat-induced epitope retrieval (HIER) method using a low pH antigen retrieval solution (Biocare Medical, Reveal Decloaker, Pacheco, CA) in a pressure cooker for 3 min. Slides were then slowly cooled and rinsed in water. Endogenous peroxidases and alkaline phosphatases were blocked by treating sections with BLOXALL® (Vector Laboratories, Burlingame, CA) for 20–30 minutes followed by two washes in tris-buffered saline with Tween (TBST) buffer. Slides were then incubated with 2.5% normal horse serum (Vector Laboratories) to block non-specific binding.

To detect the macrophage marker CD68, tissue sections were incubated overnight with a mouse monoclonal antibody (anti-CD68 Monoclonal Antibody, clone KP1, eBioscience^TM^, Life Technologies, Carlsbad, CA) at a dilution of 1/250 at 40 °C. Primary antibodies were detected by treating slides with a commercial secondary detection reagent (ImmPRESS® HRP Horse Anti-Mouse IgG PLUS Polymer Kit, Peroxidase, Vector Laboratories, Burlingame, CA) for 20 min followed by washing with TBST wash solution. Finally, antigens were detected with a brown Horse Radish Peroxidase (HRP) substrate (ImmPACT® DAB Substrate Kit, Peroxidase,Vector Laboratories) by incubating experimental slides for 1 min. Counterstaining was done with Meyer’s hematoxylin and slides were dehydrated by graded treatment with alcohol (50, 70, 90, and 100% respectively) followed by removal of alcohol with two treatments in xylene (3 min each). Permanent coverslips were mounted using Cytoseal™ XYL Mounting Medium (Richard-Allan Scientific® Cytoseal™ XYL Mounting Medium, Thermo Scientific, Waltham, MA). The macrophages were manually counted in the ten 400x high power fields (HPF) containing the most macrophages using an Olympus BX53 microscope and expressed as the average number of macrophages per HPF.

For spike protein detection, slides were incubated with the antibody (SARS-CoV-2 Spike Protein S1 rabbit Polyclonal Antibody, Invitrogen, Waltham, MA, catalogue # PA5-114528) at 1/1000 dilution overnight. Primary antibodies were detected by treating slides with a commercial secondary detection reagent (ImmPRESS®-AP Horse Anti-Rabbit IgG Polymer Detection Kit, Alkaline Phosphatase, Vector Laboratories, Burlingame, CA) for 20 min followed by washing with TBST solution. Antigens were detected with a red alkaline phosphatase substrate (Vector Laboratories) by incubating experimental slides for 1 min.

### Statistical analysis

Groups were compared using *t*-test, Fisher exact test, or chi-square test as appropriate. Quantstudio™ Design & Analysis Software v1.52 was used for amplification graphing and melting curve plot analysis. All other graphs and analysis were performed using GraphPad Prism 9 software (GraphPad Prism Software, San Diego, CA). *P* values less than 0.05 were considered significant. All statistical tests were two-sided.

### Reporting summary

Further information on research design is available in the Nature Research Reporting Summary linked to this article.

### Supplementary information

Reporting summary

Supplemental Information

## Data Availability

All data upon which conclusions are drawn are included in the manuscript or in the supplemental information file provided.

## References

[1] McColl ER (2023). COVID-19 vaccines and the virus: impact on drug metabolism and pharmacokinetics. Drug Metab. Dispos..

[2] Watson OJ (2022). Global impact of the first year of COVID-19 vaccination: a mathematical modelling study. Lancet Infect. Dis..

[3] Kwon S, Kwon M, Im S, Lee K, Lee H (2022). mRNA vaccines: the most recent clinical applications of synthetic mRNA. Arch. Pharm. Res..

[4] Szebeni J (2022). Applying lessons learned from nanomedicines to understand rare hypersensitivity reactions to mRNA-based SARS-CoV-2 vaccines. Nat. Nanotechnol..

[5] Andries O (2015). N(1)-methylpseudouridine-incorporated mRNA outperforms pseudouridine-incorporated mRNA by providing enhanced protein expression and reduced immunogenicity in mammalian cell lines and mice. J. Control. Release.

[6] Polack FP (2020). Safety and efficacy of the BNT162b2 mRNA Covid-19 vaccine. N. Engl. J. Med..

[7] Baden LR (2021). Efficacy and safety of the mRNA-1273 SARS-CoV-2 vaccine. N. Engl. J. Med..

[8] Oster ME (2022). Myocarditis cases reported after mRNA-based COVID-19 vaccination in the US from December 2020 to August 2021. J. Am. Med. Assoc..

[9] Montgomery J (2021). Myocarditis following immunization with mRNA COVID-19 vaccines in members of the US military. JAMA. Cardiol..

[10] Klein NP (2021). Surveillance for adverse events after COVID-19 mRNA vaccination. J. Am. Med. Assoc..

[11] Barda N (2021). Safety of the BNT162b2 mRNA Covid-19 vaccine in a nationwide setting. N. Engl. J. Med..

[12] Li X (2021). Characterising the background incidence rates of adverse events of special interest for covid-19 vaccines in eight countries: multinational network cohort study. Br. Med. J..

[13] García-Grimshaw M (2021). Neurologic adverse events among 704,003 first-dose recipients of the BNT162b2 mRNA COVID-19 vaccine in Mexico: a nationwide descriptive study. Clin. Immunol..

[14] Patone M (2021). Neurological complications after first dose of COVID-19 vaccines and SARS-CoV-2 infection. Nat. Med..

[15] Trougakos IP (2022). COVID-19 mRNA vaccine-induced adverse effects: unwinding the unknowns. Trends Mol. Med..

[16] European Medicines Agency. Assessment Report EMA/707383/2020 Corr.1*: Comirnaty COVID-19 MRNA Vaccine (Nucleoside-Modified), Amsterdam, The Netherlands (2021).

[17] European Medicines Agency. Assessment Report EMA/15689/2021 Corr.1*: Covid-19 Vaccine Moderna, Amsterdam, The Netherlands (2021).

[18] Röltgen K (2022). Immune imprinting, breadth of variant recognition, and germinal center response in human SARS-CoV-2 infection and vaccination. Cell.

[19] Fertig TE (2022). Vaccine mRNA can be detected in blood at 15 days post-vaccination. Biomedicines.

[20] Ogata AF (2022). Circulating severe acute respiratory syndrome coronavirus 2 (SARS-CoV-2) vaccine antigen detected in the plasma of mRNA-1273 vaccine recipients. Clin. Infect. Dis..

[21] Bansal S (2021). Cutting edge: circulating exosomes with COVID spike protein are induced by BNT162b2 (Pfizer-BioNTech) vaccination prior to development of antibodies: a novel mechanism for immune activation by mRNA vaccines. J. Immunol..

[22] Bearse M (2021). Factors associated with myocardial SARS-CoV-2 infection, myocarditis, and cardiac inflammation in patients with COVID-19. Mod. Pathol..

[23] Stein SR (2022). SARS-CoV-2 infection and persistence in the human body and brain at autopsy. Nature.

[24] Lederer K (2022). Germinal center responses to SARS-CoV-2 mRNA vaccines in healthy and immunocompromised individuals. Cell.

[25] Wu YW (2016). Ionic liquids impact the bioenergy feedstock-degrading microbiome and transcription of enzymes relevant to polysaccharide hydrolysis. mSystems.

[26] Hsieh CL (2020). Structure-based design of prefusion-stabilized SARS-CoV-2 spikes. Science.

[27] Xia X (2021). Domains and functions of spike protein in Sars-Cov-2 in the context of vaccine design. Viruses.

[28] Remmelink M (2020). Unspecific post-mortem findings despite multiorgan viral spread in COVID-19 patients. Crit. Care.

[29] Granados-Riveron JT, Aquino-Jarquin G (2021). Engineering of the current nucleoside-modified mRNA-LNP vaccines against SARS-CoV-2. Biomed. Pharmacother..

[30] Johnson PA, Jaffer FA, Neilan TG, Shepard JAO, Stone JR (2006). Case records of the Massachusetts General Hospital. Case 34-2006. A 72-year-old woman with nausea followed by hypotension and respiratory failure. N. Engl. J. Med..

[31] Stone JR (2012). Pathology of myocardial infarction, coronary artery disease, plaque disruption, and the vulnerable atherosclerotic plaque. Diagn. Histopathol..

