# Supplementary material for: Duration of SARS-CoV-2 mRNA vaccine persistence and factors associated with cardiac involvement in recently vaccinated patients
Source: NPJ Vaccines. 2023 Sep 27;8:141. doi: 10.1038/s41541-023-00742-7 (PMC10533894; doi:10.1038/s41541-023-00742-7)
Supplement: Supplementary file 1 — Reporting summary [file 41541_2023_742_MOESM1_ESM.pdf]

nature portfolio

Corresponding author(s): James R. Stone

Last updated by author(s): Sep 5, 2023

Reporting Summary

Nature Portfolio wishes to improve the reproducibility of the work that we publish. This form provides structure for consistency and transparency in reporting. For further information on Nature Portfolio policies, see our Editorial Policies and the Editorial Policy Checklist.

Statistics

For all statistical analyses, confirm that the following items are present in the figure legend, table legend, main text, or Methods section.

n/a	Confirmed	
☐	☒	The exact sample size (n) for each experimental group/condition, given as a discrete number and unit of measurement
☐	☒	A statement on whether measurements were taken from distinct samples or whether the same sample was measured repeatedly
☐	☒	The statistical test(s) used AND whether they are one- or two-sided<br>*Only common tests should be described solely by name; describe more complex techniques in the Methods section.*
☐	☒	A description of all covariates tested
☐	☒	A description of any assumptions or corrections, such as tests of normality and adjustment for multiple comparisons
☐	☒	A full description of the statistical parameters including central tendency (e.g. means) or other basic estimates (e.g. regression coefficient) AND variation (e.g. standard deviation) or associated estimates of uncertainty (e.g. confidence intervals)
☐	☒	For null hypothesis testing, the test statistic (e.g. F, t, r) with confidence intervals, effect sizes, degrees of freedom and P value noted<br>*Give P values as exact values whenever suitable.*
☒	☐	For Bayesian analysis, information on the choice of priors and Markov chain Monte Carlo settings
☒	☐	For hierarchical and complex designs, identification of the appropriate level for tests and full reporting of outcomes
☒	☐	Estimates of effect sizes (e.g. Cohen's d, Pearson's r), indicating how they were calculated

Our web collection on statistics for biologists contains articles on many of the points above.

Software and code

Policy information about availability of computer code

Data collection	Quantstudio™ Design & Analysis Software v1.52
Data analysis	Quantstudio™ Design & Analysis Software v1.52, GraphPad Prism 9

For manuscripts utilizing custom algorithms or software that are central to the research but not yet described in published literature, software must be made available to editors and reviewers. We strongly encourage code deposition in a community repository (e.g. GitHub). See the Nature Portfolio guidelines for submitting code & software for further information.

Data

Policy information about availability of data

All manuscripts must include a data availability statement. This statement should provide the following information, where applicable:

- Accession codes, unique identifiers, or web links for publicly available datasets
- A description of any restrictions on data availability
- For clinical datasets or third party data, please ensure that the statement adheres to our policy

All data upon which conclusions are made are included in the manuscript or in the supplemental information file provided.

Research involving human participants, their data, or biological material

Policy information about studies with human participants or human data. See also policy information about sex, gender (identity/presentation), and sexual orientation and race, ethnicity and racism.

Reporting on sex and gender	The findings are not limited to only one biologic sex. Biologic sex was not considered in the study design. Sex refers to the sex assigned at birth which in all cases was confirmed at autopsy by examination of internal and external organs. The requirement for patient consent for this project was waived by the IRB, and patient consent was not obtained. The study involved a total of 15 male decedents and 10 female decedents. The detection of vaccine in the heart was not associated with sex.
Reporting on race, ethnicity, or other socially relevant groupings	Race was categorized as white, black, or unknown based on the medical record. Confounding variables regarding race were not controlled for due to the small sample size.
Population characteristics	The vaccinated patients entailed 20 patients with a mean age of 64, a mean BMI of 26 and histories of coronary artery disease (33%), hypertension (78%), hyperlipidemia (72%), diabetes mellitus (33%), autoimmune disease (17%), and smoking (50%).
Recruitment	The patients recruited all underwent autopsy performed at Massachusetts General Hospital between January 2021 and February 2022. Inclusion criteria were the ability to collect fresh tissue, a clear history of either vaccination or no vaccination, and a post-mortem interval ≤ 60 hours. Patients with a history of SARS-CoV-2 infection were excluded.
Ethics oversight	Mass General Brigham IRB

Note that full information on the approval of the study protocol must also be provided in the manuscript.

Field-specific reporting

Please select the one below that is the best fit for your research. If you are not sure, read the appropriate sections before making your selection.

☒ Life sciences ☐ Behavioural & social sciences ☐ Ecological, evolutionary & environmental sciences

For a reference copy of the document with all sections, see nature.com/documents/nr-reporting-summary-flat.pdf

Life sciences study design

All studies must disclose on these points even when the disclosure is negative.

Sample size	Samples size was determined based on the number of autopsies that met the study criteria.
Data exclusions	No data were excluded.
Replication	All PCR-positive samples were confirmed by sequencing.
Randomization	Samples were allocated into groups based on the presence or absence of vaccine detected by RT-PCR. The number samples positive for vaccine were small and covariates were not controlled.
Blinding	The investigator performing the vaccine RT-PCR was blinded to the presence of myocardial injury and the macrophage counts.

Reporting for specific materials, systems and methods

We require information from authors about some types of materials, experimental systems and methods used in many studies. Here, indicate whether each material, system or method listed is relevant to your study. If you are not sure if a list item applies to your research, read the appropriate section before selecting a response.

Materials & experimental systems

n/a	Involved in the study
	☒ Antibodies
☒	☐ Eukaryotic cell lines
☒	☐ Palaeontology and archaeology
☒	☐ Animals and other organisms
	☒ Clinical data
☒	☐ Dual use research of concern
☒	☐ Plants

Methods

n/a	Involved in the study
☒	☐ ChIP-seq
☒	☐ Flow cytometry
☒	☐ MRI-based neuroimaging

Antibodies

Antibodies used

SARS-CoV-2 Spike Protein S1 rabbit Polyclonal Antibody, Invitrogen, Waltham, MA, catalogue # PA5-114528
Anti-CD68 Monoclonal Antibody, clone KP1, eBioscienceTM, Life Technologies, Carlsbad, CA, catalogue # 14-0688-82

Validation

Lung tissue from patients who died of severe COVID-19 was used as a positive control for both antibodies.

Clinical data

Policy information about clinical studies
All manuscripts should comply with the ICMJE guidelines for publication of clinical research and a completed CONSORT checklist must be included with all submissions.

Clinical trial registration

Not applicable, this was not a clinical trial.

Study protocol

Not applicable, this was not a clinical trial

Data collection

The patients recruited all underwent autopsy performed at Massachusetts General Hospital between January 2021 and February 2022. Clinical data was collected following the autopsies.

Outcomes

Study outcomes were the presence or absence of vaccine in the tissues as determined by RT-PCR.
