# Supplementary material for: Duration of SARS-CoV-2 mRNA vaccine persistence and factors associated with cardiac involvement in recently vaccinated patients
Source: NPJ Vaccines. 2023 Sep 27;8:141. doi: 10.1038/s41541-023-00742-7 (PMC10533894; doi:10.1038/s41541-023-00742-7)
Supplement: Supplementary file 2 — Supplemental Information [file 41541_2023_742_MOESM2_ESM.pdf]

Duration of SARS-CoV-2 mRNA Vaccine Persistence

and Factors Associated with Cardiac Involvement in Recently Vaccinated Patients

Aram J. Krauson[1], Faye Victoria C. Casimero[1,2], Zakir Siddiquee[1], James R. Stone[1,2]

[1]Department of Pathology, Massachusetts General Hospital, Boston, MA

[2]Department of Pathology, Harvard Medical School, Boston, MA

Supplemental Information

Table of Contents

Page 2: Supplementary Table 1. Characteristics of the vaccinated patients and non-vaccinated control patients

Page 3: Supplementary Table 2. Vaccine administrations

Page 4: Supplementary Table 3. Primer sets for validation of detected vaccines

Page 5: Supplementary Table 4. RT-PCR validation of vaccine-detected samples

Page 6: Supplementary Table 5. Quality control primer/probe sets

Page 7: Supplementary Figure 1. Cardiac macrophages in the patients with vaccine detected in the heart

Page 8: Supplementary Figure 2. Double-stranded DNA templates used as positive controls

Page 9: Supplementary Figure 3. Primer efficiency of selected primers

Page 10: Supplementary References

Supplementary Table 1. Characteristics of the vaccinated patients and non-vaccinated control patients.

Variable	Vaccinated	Control	P
Patients (N)	20	5	
Age (years), mean ± SD	64 ± 16	57 ± 18	0.44[1]
Male, n (%)	12 (60)	3 (60)	1.00[2]
Race			0.07[2]
White, n (%)	18 (90)	3 (60)	
Black or African American, n (%)	0 (0)	2 (40)	
Unknown, n (%)	2 (10)	0 (0)	
BMI (kg/m^2), mean ± SD	26 ± 9	30 ± 10	0.37[1]
History of CAD, n/total (%)	6/18 (33)	1 (20)	1.00[2]
History of hypertension, n/total (%)	14/18 (78)	4 (80)	1.00[2]
History of hyperlipidemia, n/total (%)	13/18 (72)	3 (60)	0.62[2]
History of diabetes mellitus, n/total (%)	6/18 (33)	2 (40)	1.00[2]
History of autoimmune disease, n/total (%)	3/18 (17)	0 (0)	1.00[2]
Current/former smoker, n/total (%)	9/18 (50)	2/4 (50)	1.00[2]
Post-mortem interval (hours), mean ± SD	23 ± 11	30 ± 20	0.52[1]
Heart weight (grams), mean ± SD	469 ± 164 (n=19)	470 ± 181	0.99[1]
Left ventricular wall thickness (cm), mean ± SD	1.3 ± 0.2	1.2 ± 0.3	0.68[1]
Pericarditis, n (%)	3 (15)	1 (20)	1.00[2]
Severe CAD (≥75% stenosis), n/total (%)	6/19 (32)	1/5 (20)	1.00[2]
Any acute or recent myocardial injury, n (%)	11 (55)	1 (20)	0.32[2]
Cause of Death, n (%)			0.99[3]
Coronary artery disease	4 (20)	1 (20)	
Malignancy	4 (20)	1 (20)	
Cardiomyopathy	3 (15)	1 (20)	
Infection	3 (15)	1 (20)	
Neurodegenerative disease	3 (15)	1 (20)	
Hemorrhage	2 (10)	0 (0)	
Cirrhosis	1 (5)	0 (0)	

[1]t test.
[2]Fisher exact test.
[3]Chi-square test.
BMI body mass index, *CAD* coronary artery disease.

Supplementary Table 2. Vaccine administrations.

Patient #	Vaccine	Days From First Dose	Days From Second Dose	Days From Third Dose	Days From Last Dose
1	BNT162b2	21	1	N/A	1
2	mRNA-1273	9	N/A	N/A	9
3	BNT162b2	18	N/A	N/A	18
4	mRNA-1273	22	N/A	N/A	22
5	BNT162b2	26	N/A	N/A	26
6	BNT162b2	Unknown	17	N/A	17
7	BNT162b2	12	N/A	N/A	12
8	BNT162b2	40	19	N/A	19
9	mRNA-1273	108	78	N/A	78
10	BNT162b2	Unknown	2	N/A	2
11	BNT162b2	27	3	N/A	3
12	BNT162b2	Unknown	68	N/A	68
13	BNT162b2	82	N/A	N/A	82
14	mRNA-1273	55	15	N/A	15
15	BNT162b2	108	86	N/A	86
16	BNT162b2	178	149	N/A	149
17	mRNA-1273	182	154	N/A	154
18	mRNA-1273	173	145	N/A	145
19	mRNA-1273	284	256	9	9
20	BNT162b2	340	319	38	38

N/A not applicable.

Supplementary Table 3. Primer sets for validation of detected vaccines.

Target	Primers	Primer Sequence 5' – 3'	NTs
BNT162b2	Forward	CAGAACACAGCTGCCTCCAG	60-563
	Reverse	TTCTTGAAGTTGCCCTGCTT	
	Forward	CCAACGTGGTCATCAAAGTG	371-1544
	Reverse	GGAACAGCCGGTACAGGTAA	
	Forward	GGTGCAGATCGACAGACTGA	2970-3650
	Reverse	CAGATGTACCAGGGCCACTT	
mRNA-1273	Forward	CTGTTCCTGCCCTTCTTCAGC	160-565
	Reverse	GGTTCTTGAAGTTGCCCTGCTTG	
	Forward	CTGTTCCTGCCCTTCTTCAGC	160-1176
	Reverse	GAAGCACAGGTCGTTCAGCTTGG	

Nucleotide (NT) sequences are numbered starting at the start codon of the signal peptide sequence of each designed mRNA vaccine (Supplementary Reference 1). Annealing temperature was set at 60 °C for all sets at an elongation time of 60 seconds per 1000 bps.

Supplementary Table 4. RT-PCR validation of vaccine-detected samples.

Target	Patient #	Tissue	NTs Sequenced
BNT162b2	1	ALN	84 – 566; 3004 – 3620
	5	ALN	410 – 1461
	6	ALN	2982 – 3650
	7	ALN	416 – 1448
	10	ALN	60 – 563; 418 – 1463; 3016 – 3641
	11	ALN	104 – 501
	7	LV	102 – 518
	10	LV	60 – 563; 2982 – 3641
	8	RV	105 – 563
	10	RV	102 – 392
mRNA-1273	4	ALN	172 – 552
	14	ALN	208 – 579; 887 – 1119

Three primer sets were randomly or in combination tested with samples with detected BNT162b2, whereas two sets were tested with the two mRNA-1273 positive samples. All segments listed in the NTs sequenced column contain 400+ nucleotides outside the RT-qPCR amplicon sequence. *ALN* axillary lymph nodes, *LV* left ventricle, *RV* right ventricle.

Supplementary Table 5. Quality control primer/probe sets.

Target	Primers	Primer Sequence 5’ – 3’	End Concentration	Annealing Temp. (°C)
ACTB	Forward	GCACAGAGCCTCGCCTTTG	300 nM	60
	Reverse	ATCCATGGTGAGCTGGCG		
GAPDH	Forward	AGCCACATCGCTCAGACAC	300 nM	60
	Reverse	GCCCAATACGACCAAATCC		
E Sarbeco	Forward	ACAGGTACGTTAATAGTTAATAGCGT	400 nM	58 [1]
	Reverse	ATATTGCAGCAGTACGCACACA		
	Probe	ACACTAGCCATCCTTACTGCGCTTCG	200 nM	

[1] Supplementary Reference 2.

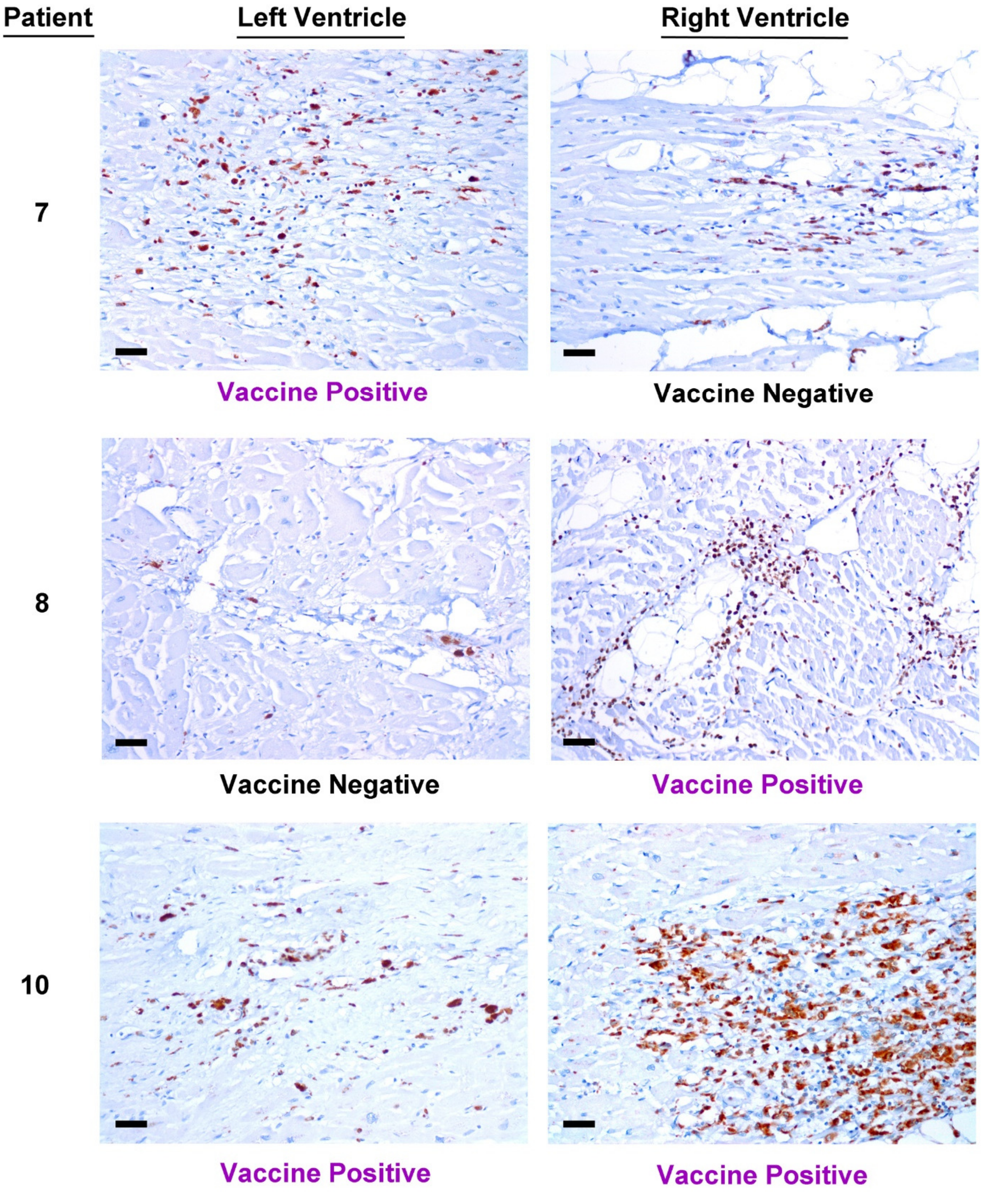

Supplementary Figure 1. Cardiac macrophages in the patients with vaccine detected in the heart
Depicted are immunohistochemical stains for the macrophage marker CD68 (brown color) in the three patients with vaccine detected in the heart for both the left ventricle (left) and right ventricle (right). Vaccine was detected by PCR in four of the six ventricles as indicated. Scale bars indicate 40 microns.

a

GGCATCGGAGTGACCCAGAATGTGCTGTACGAGAACCAGAAGCTGATCGCCAACCAGTTCAACAGC
GCCATCGGCAAGATCCAGGACAGCCTGAGCAGCACAGCAAGCGCCCTGGGAAAGCTGCAGGACGT
GGTCAACCAGAATGCCCAGGCACTGAACACCCTGGTCAAGCAGCTGTCCTCCAACTTCGGCGCCAT
CAGCTCTGTGCTGAACGATATCCTGAGCAGACTGGACCCTCCTGAGGCCGAGGTGCAGATCGACAG
ACTGATCACAGGCAGACTGCAGAGCCTCCAGACATACGTGACCCAGCAGCTGATCAGAGCCGCCGA
GATTAGAGCCTCTGCCAATCTGGCCGCCACCAAGATGTCTGAGTGTGTGCTGGGCCAGAGCAAGAG
AGTGGACTTTTGCGGCAAGGGCTACCACCTGATGAGCTTCCCTCAGTCTGCCCCT

FWD Primer: GGTGCAGATCGACAGACTGA

REV Primer: GGGAAGCTCATCAGGTGGTA

Probe: TACGTGACCCAGCAGCTGATCAGAGCC

b

ACAACACCTTCGTGAGCGGCAACTGCGACGTGGTGATCGGCATCGTGAACAACACCGTGTACGATC
CCCTGCAGCCCGAGCTGGACAGCTTCAAGGAGGAGCTGGACAAGTACTTCAAGAATCACACCAGCC
CCGACGTGGACCTGGGCGACATCAGCGGCATCAACGCCAGCGTGGTGAACATCCAGAAGGAGATC
GATCGGCTGAACGAGGTGGCCAAGAACCTGAACGAGAGCCTGATCGACCTGCAGGAGCTGGGCAA
GTACGAGCAGTACATCAA

FWD Primer: AGCTGGACAGCTTCAAGGAG

REV Primer: GGCTCTCGTTCAGGTTCTTG

Probe: ACGCCAGCGTGGTGAACATCC

Supplementary Figure 2. Double-stranded DNA templates used as positive controls for vaccine detection assays

a. Positive control dsDNA fragment for BNT162b2 with forward and reverse primer sequences below and underlined within the fragment's sequence. The Taqman probe is listed below and highlighted in the control sequence. The vaccine's proline substitutions (in red) are included in the dsDNA control. b. The mRNA-1273 detection assay dsDNA fragment control sequence is shown with both listed primers (underlined) and probe (highlighted yellow) used.

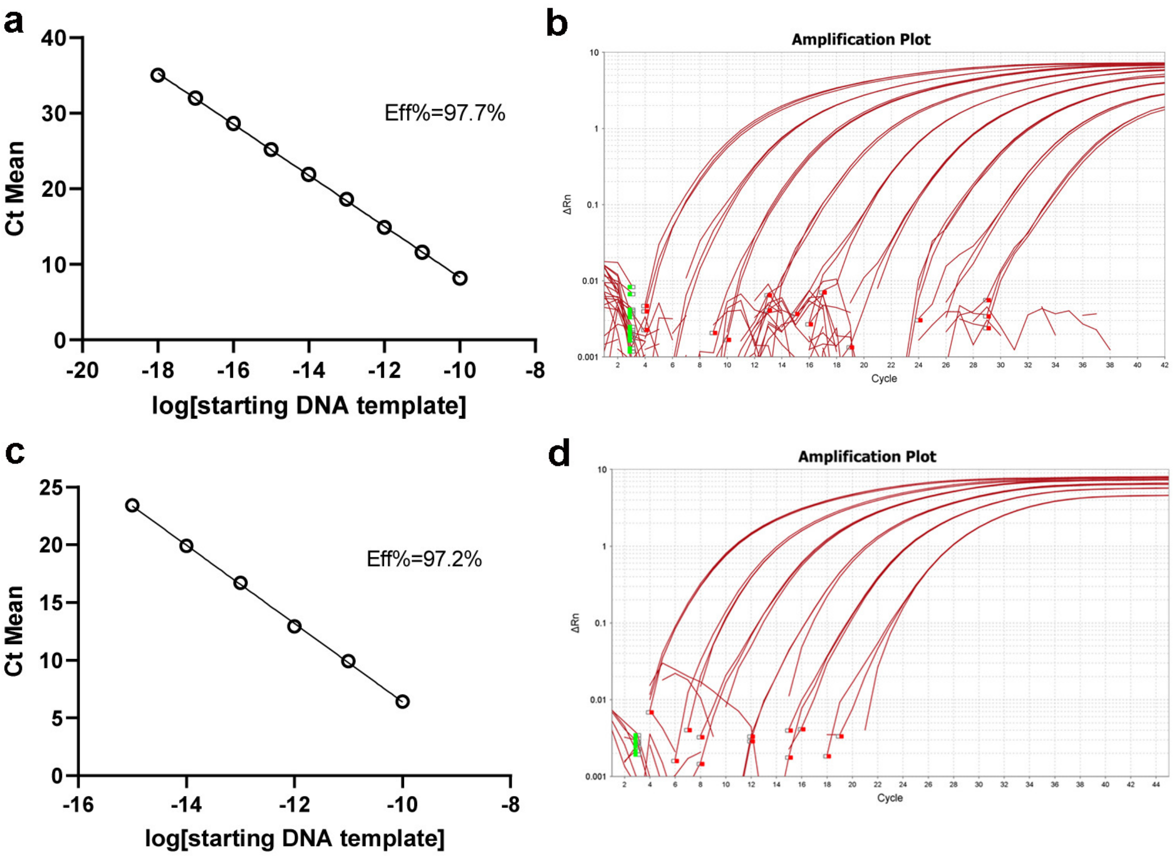

Supplementary Figure 3. Primer efficiency for two SARS-Cov2 mRNA vaccine-detection assays
The primer efficiency for each SARS-Cov2 vaccine detection assay was tested using serially diluted dsDNA fragment stocks in triplicate**.** a. b. Trendline outcome and amplification plot is shown for BNT162b2 vaccine RT-qPCR assay. Calculated primer efficiency is 97.7%, slope = -3.378, R^2 =0.9998. c. d. Primer efficiency graph with trendline and amplification plot for the mRNA-1273 detection assay. The primer set had a primer efficiency of 97.2%, slope = -3.392, R^2 = 0.9995.

Supplementary References

1. Granados-Riveron, J. T. & Aquino-Jarquin, G. Engineering of the current nucleoside-modified mRNA-LNP vaccines against SARS-CoV-2. *Biomed. Pharmacother.* **142**, 111953 (2021).

2. Remmelink, M. et al. Unspecific post-mortem findings despite multiorgan viral spread in COVID-19 patients. *Crit. Care* **24,** 495 (2020).
